# Correlation of Circulating Acid-Labile Subunit Levels with Insulin Sensitivity and Serum LDL Cholesterol in Patients with Type 2 Diabetes: Findings from a Prospective Study with Rosiglitazone

**DOI:** 10.1155/2014/917823

**Published:** 2014-05-22

**Authors:** Ying-Chuen Lai, Hung-Yuan Li, Ta-Jen Wu, Chi-Yuan Jeng, Lee-Ming Chuang

**Affiliations:** ^1^Department of Internal Medicine, National Taiwan University Hospital, Yun-Lin Branch, Yun-Lin, Taiwan; ^2^Department of Internal Medicine, National Taiwan University Hospital, 7 Chung-Shan South Road, Taipei 10002, Taiwan; ^3^Department of Internal Medicine, National Cheng Kung University Hospital, Tainan, Taiwan; ^4^Lin Shin Hospital, Taichung, Taiwan; ^5^Graduate Institute of Clinical Medicine, National Taiwan University School of Medicine, Taipei, Taiwan

## Abstract

Silencing of acid-labile subunit (ALS) improved glucose metabolism in animal models. The aim of this study is to evaluate the effects of rosiglitazone (RSG) on ALS levels in individuals with type 2 diabetes. A randomized, double-blind, placebo-controlled trial was conducted. Subjects with type 2 diabetes mellitus were randomly distributed to an RSG-treated (*n* = 30) or a placebo (*n* = 31) group. Patients were evaluated prior to treatment at baseline and at 12 and 24 weeks after treatment. At baseline, ALS levels were negatively associated with low-density lipoprotein cholesterol (LDLc) levels and homeostatic model assessment version 2 insulin sensitivity (HOMA2-%S). Over 24 weeks, there was a significantly greater reduction in ALS levels in the nonobese RSG-treated individuals than placebo-treated group. The effect of RSG on ALS was not significant in obese individuals. Fasting plasma glucose and hemoglobin A1c were reduced, but total cholesterol and LDLc were increased, in patients on RSG. Change in ALS levels predicted changes in total cholesterol and HOMA2-%S over time. This study suggested a BMI-dependent effect of RSG treatment on ALS levels. Reduction of ALS by RSG increases the risk of atherosclerosis in individuals with type 2 diabetes.

## 1. Introduction

Acid-labile subunit (ALS) is a 63.3 kDa glycoprotein that is encoded by the* IGFALS* gene at the chromosomal location 16p13.3. ALS is secreted by the liver and found in the circulation, but it is also expressed in the lung, intestine, heart, kidney, and adipose tissues [1]. ALS functions to stabilize insulin-like growth factor (IGF) by forming a 150 kDa ternary complex consisting of ALS, IGF-1, and IGF-binding protein (IGFBPs) 3 or 5, resulting in prolonged retention of IGF-1 in the circulation [2]. Growing evidence supports a functional link between ALS and insulin sensitivity and glucose metabolism. Mice lacking the* IGFALS* gene (ALSKO) were leaner and had an increased percentage of fat mass as compared with wild-type mice. Furthermore, the glucose clearance rate was faster in the ALSKO mice compared with wild-type controls [3, 4]. Drosophilae with silenced dALS, encoding the fly ortholog of vertebrate ALS, were also determined to have lower circulating glucose levels [5].

Peroxisome proliferator-activated receptor (PPAR*γ*), a lipid-activated nuclear receptor, improves insulin sensitivity and increases the expression of adiponectin [6]. Activation of PPAR*γ* influences cholesterol metabolism by enhancing the reverse cholesterol transport pathway through the efflux of cholesterol to lipid-poor apolipoprotein A-I [7]. The PPAR*γ* agonist rosiglitazone (RSG) is one of the thiazolidinedione drugs that has been used for the treatment of type 2 diabetes. In clinical studies, RSG treatment lowered hemoglobin A1c (HbA1c) and increased high-density lipoprotein cholesterol (HDLc) and low-density lipoprotein cholesterol (LDLc) levels [8–10].

Recent studies have shown that PPAR*γ* agonists regulate the IGF system [11–13]. For example, RSG decreased expression of IGF-1 and increased IGFBP-1 in cell culture experiments and in humans [11–13]. We previously documented that expression of ALS is upregulated during adipocyte differentiation in cultured mouse 3T3-L1 preadipocytes. In fully differentiated 3T3-L1 adipocytes, we demonstrated that ALS messenger ribonucleic acid was repressed after treatment with RSG for 24 hours [14]. Whether a decrease of serum ALS is related to insulin sensitivity or dyslipidemia is not known. The purpose of the present study was to investigate the relationship between changes in ALS levels and metabolic changes upon RSG treatment in patients with type 2 diabetes.

## 2. Methods

### 2.1. Study Population

This protocol was approved by the Human Research Committee of the National Taiwan University College of Medicine, National Taiwan University Hospital, and Taiwan Department of Health and is registered in the Clinical Trials Protocol Registration System (NCT01706211) and was performed in accordance with the Declaration of Helsinki. Written informed consent was obtained from each participant.

**Figure 1:**
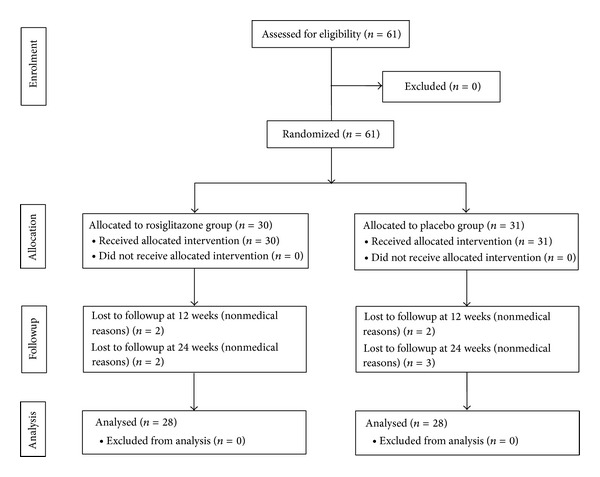
Flowchart of the study design.

A double-blind, placebo-controlled, parallel-group comparative study was conducted between 1999 and 2000 to evaluate the effects of RSG (BRL 49653C) and concurrent sulfonylurea therapy [15] (Figure 1). Patient inclusion criteria were men or women between 30 and 80 years, patients with type 2 diabetes mellitus defined by the World Health Organization (WHO) criteria and having a poor glycemic control with HbA1c levels ≥7.5% (58 mmol/mol) and a fasting plasma glucose level of ≤15.0 mmol/L at the screening. Exclusion criteria were other severe medical problems and microvascular complications that required immediate medical attention. In addition, patients that were stable on sulfonylurea therapy for at least 2 months before the screening visit were recruited for the study. During the screening visit, patients entered a single-blind, 4-week placebo/sulfonylurea run-in period to establish baseline characteristics. Patients were then randomized for the double-blind phase. RSG and matching placebo tablets were supplied by SmithKline Beecham Pharmaceuticals, UK. Each patient received 2 tablets of either RSG (Avandia, 2 mg/tablet) or placebo (control) in a dose regimen of 1 tablet twice daily for 24 weeks. Participants had scheduled visits every 4 weeks. Blood samples were collected during the study and were stored until the insulin and glucose metabolism parameters were measured.

### 2.2. Anthropometric and Biochemical Measurements

Body weight, height, blood pressure, and heart rate were measured. Body mass index (BMI) was calculated as weight (kg)/height (m^2^). As the WHO suggested population-specific cut-off points for BMI to identify those with increased risk for type 2 diabetes and cardiovascular disease [16], we adopted a consensus criteria for defining overweight (BMI ≥ 24–26.9 kg/m^2^) and obesity (BMI ≥ 27 kg/m^2^) by the Department of Health, Taiwan, according to a comparative study for Asians [17]. Fasting plasma glucose, serum total cholesterol, triglyceride, LDLc, and HDLc levels were measured from blood drawn after an overnight fast (Hitachi 7250 Special; Hitachi, Tokyo, Japan). Fasting plasma insulin concentration was measured on an automatic analyzer using a microparticle-based enzyme immunoassay (Abbott AxSYM system, Abbott Laboratories, Abbott Park, IL, USA). HbA1c was measured using a DCA2000 analyzer (Bayer Sankyo, Tokyo, Japan). The homeostasis model assessment was applied as described previously [18, 19]. For the measurement of ALS serum levels, enzyme-linked immunosorbent assays were performed with commercial kits (Mediagnost, Reutlingen, Germany) [20]. Interassay variance was ≤8%, intra-assay variance was ≤6.8%, and the kit sensitivity was 0.23 mU/mL. Study outcomes were assessed at the 12- and 24-week visits and compared to those at baseline.

### 2.3. Sample Size Consideration

The primary endpoint of the study was the treatment-induced change in HbA1c from baseline to week 24. A sample size of 52 patients (26 for each treatment group) was determined after considering a 20% dropout rate and a 90% power to detect a difference of 1.1 in HbA1c between treatment groups (if the standard deviation of the response is 1.1, based on an *α* of 0.05 [two-sided]). There are no previous clinical trials to evaluate the effect of RSG on serum ALS levels. A minimum sample size of 20 participants per group is required for analysis in a pilot study [21].

### 2.4. Statistical Analysis

Descriptive data are presented as means ± standard deviations or as percentages for categorical variables. Student's *t*-test was used to compare the baseline characteristics. Fisher's exact tests were used to compare categorical variables. Fasting plasma insulin, serum triglyceride, ALS levels, homeostatic model assessment version 2 insulin sensitivity (HOMA2-%S), and homeostatic model assessment version 2 beta-cell function (HOMA2-%B) were not normally distributed, so the results were log-transformed for analysis. Correlations between serum ALS levels and metabolic measures were examined by linear regression. Multivariate regression models were created to evaluate the relationship between changes in ALS levels, HOMA2-%S, and LDLc levels due to treatment. Metabolic variables correlated to baseline ALS levels with *P* < 0.15 were used as covariates. The general linear mixed model for repeated measures (SAS software version 9.2, SAS Institute, Cary, NC) was used to assess the effect of RSG treatment on the 24-week change in serum ALS levels and metabolic measures over the course of the trial. The multilevel models included treatment, time, treatment × time, age, gender, LDLc, and HOMA2-%S. With the same statistical models, an interaction term of ALS × time was included to estimate changes in total cholesterol over time. Changes in ALS levels between the RSG and placebo groups in nonobese and obese subgroup analysis were assessed with the Wilcoxon rank-sum test. The analyses were carried out without and with imputation of missing values, using the last observation carried forward method. A *P* value of <0.05 was considered statistically significant.

## 3. Results

### 3.1. Subject Characteristics

Of the 61 enrolled subjects, 30 were randomly assigned to the RSG treatment group and 31 were assigned to the placebo group. The mean patient age was 58 ± 9.24 years, and 57.4% were women. Most subjects were hyperglycemic with a mean fasting plasma glucose level of 11.05 ± 2.84 mmol/L and an HbA1c level of 9.83 ± 1.51% (84 ± 16.5 mmol/mol). Over half of the patients (42/61, 68.9%) had a BMI above 24 kg/m^2^. As shown in Table 1, both groups were matched for age, sex, BMI, blood glucose concentration, HOMA2-%S, HOMA2-%B, and lipid profile, except systolic blood pressure. Serum ALS levels were not significantly different between the RSG-treated group and the placebo group at baseline.

### 3.2. Correlations between ALS Levels and Metabolic Traits at Baseline

**Figure 2:**
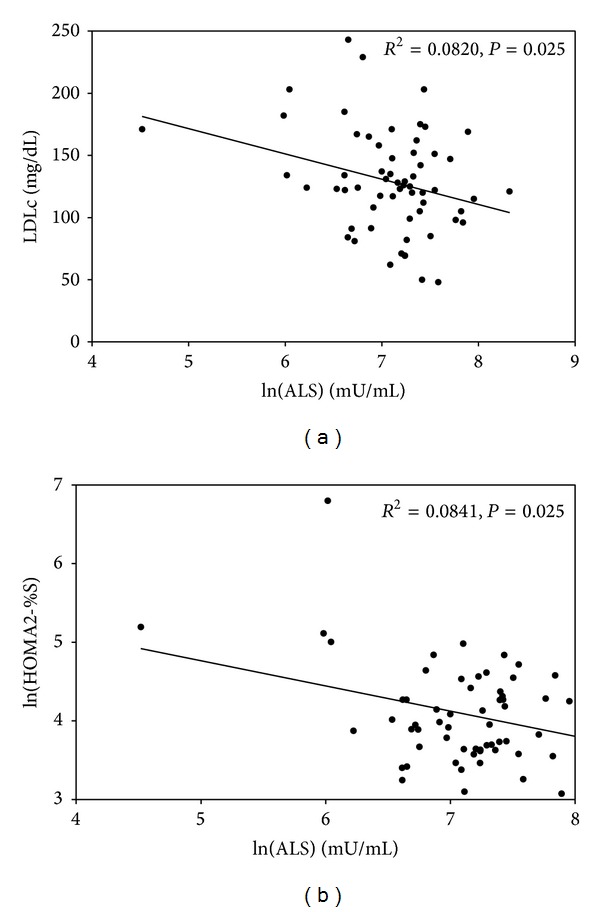
Relationships between acid-labile subunit (ALS) levels and metabolic parameters. The correlations of ALS levels with low-density lipoprotein cholesterol (LDLc) concentrations (a) and homeostatic model assessment version 2 insulin sensitivity (HOMA2-%S) (b) were significant at baseline.

Women had higher ALS levels than men (1553.5 ± 695.7 versus 1142.6 ± 583.2; *P* = 0.008). Baseline serum ALS levels were negatively correlated with body height (*r* = −1.785, *P* = 0.041), LDLc (*r* = −0.156, *P* = 0.025), and the HOMA2-%S (*r* = −0.262, *P* = 0.025) and positively correlated with fasting plasma insulin (*r* = 0.259; *P* = 0.022). Since ALS has been shown to decrease with increasing age [22], we added age into the adjustment. Serum LDLc and HOMA2-%S remained significantly related to serum ALS (Figure 2).

### 3.3. Effect of RSG Treatment on ALS Levels and Metabolic Traits

Five subjects (2 in the RSG-treated group and 3 in the placebo group) were lost to followup for personal or nonmedical reasons and/or had missing data of ALS levels at the 24-week endpoint.

Using separate mixed models, we found that the RSG-treated group experienced progressive improvements in HbA1c levels, fasting plasma glucose levels, and the HOMA2-%B compared with the placebo group over the 24-week study period. The RSG-treated group had decreased HOMA-insulin resistance (HOMA-IR) as compared with the placebo group at 12 weeks (*P* = 0.005) but not at 24 weeks. At the end of the trial, HbA1c levels had decreased by 1.16% (12.7 mmol/mol), whereas body weight increased by 2.98 ± 2.09 kg, and total cholesterol and LDLc levels increased by 0.85 ± 0.81 and 0.86 ± 0.88 mmol/L, respectively, in the RSG-treated group (Table 2). Rate of LDL > 2.6 mmol/L increased 70.0% to 93.1% in RSG group (*P* = 0.042), while there are no changes in the placebo group (80.6% to 83.3%, *P* = 1.000).

**Figure 3:**
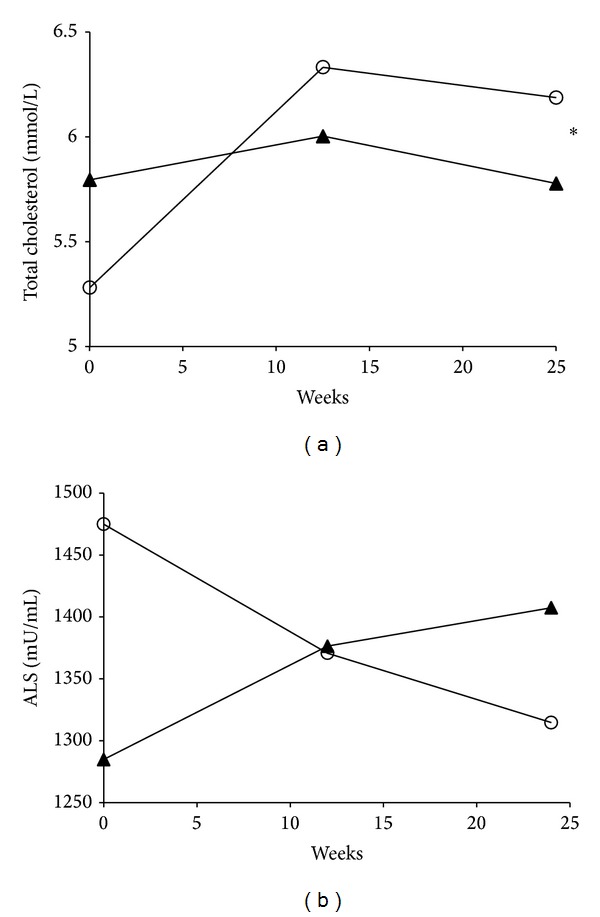
Change in total cholesterol and acid-labile subunit (ALS) in the placebo (▲) and the rosiglitazone group (○). *P_trend_ = 0.0302 for change in total cholesterol by change in ALS over time.

Owing to the fact that growth hormone (GH) secretion is blunted in obese individuals [23–25], ALS levels are influenced by obesity. We divided the study participants into nonobese (BMI < 24 kg/m^2^) and obese (BMI 24 kg/m^2^) groups. In the nonobese group, ALS levels decreased in the RSG-treated group as compared with the placebo group (Table 3). Similar results were observed with repeated measures analysis of covariance, adjusted for age, gender, LDLc, or the HOMA2-%S (*P*_trend_ = 0.0273). We evaluated the relationship between the changes in ALS levels, HOMA2-%S, and LDLc using data from 30 subjects in placebo group and 31 subjects in RSG group (Table 4). Changes in ALS levels were positively correlated with changes in HOMA2-%S after adjusting for various confounders. A further analysis using linear mixed-effects models was used to test whether the change in ALS levels could predict metabolic parameters over time. We found that the interaction term “ALS × time” independently predicted changes in total cholesterol levels (*P* = 0.0302) (Figure 3) and did not predict changes in HbA1c and HOMA2-%S.

## 4. Discussion

To the best of our knowledge, this is the first study to examine the correlation of ALS levels with metabolic phenotypes and the effect of the insulin-sensitizer RSG on ALS levels in subjects with type 2 diabetes. We found that, at baseline, ALS levels were highly correlated with age, height, fasting plasma insulin levels, HOMA2-%S, and serum LDLc concentrations. After 24 weeks, we observed a significant decrease in ALS levels in nonobese subjects with type 2 diabetes treated with RSG, as compared with the placebo group. The effect of treatment on ALS levels was not observed in obese individuals, indicating a heterogeneous response to RSG therapy according to BMI of the subjects.

Serum ALS levels were lower in men than women with type 2 diabetes. In a previous meta-analysis, it had been shown that testosterone levels were lower in men and higher in women with type 2 diabetes as compared to the healthy controls [26]. GH secretion is stimulated by testosterone. Consistently, it was reported that GH concentration was increased after testosterone therapy in hypogonadal men who had reduced GH pulse amplitude [27]. These findings might support our finding of gender difference of ALS as most liver ALS synthesis is regulated by GH.

Our results are consistent with previous observations in animals and provide the first demonstration that an increase in serum ALS levels is associated with insulin resistance in patients with type 2 diabetes. Our study showed that subjects with lower serum ALS had less insulin resistance. When considering the lower ALS levels in older people [22], we found that age-adjusted ALS remained related to insulin resistance (Figure 2). In well-defined systems, data obtained from animal experiments suggest that ALS may control insulin and glucose homeostasis. Arquier et al. first demonstrated in drosophila that ALS is involved in the regulation of carbohydrate metabolism, as carbohydrate levels in hemolymphs were increased by 25% in groups overexpressing ALS and were decreased by 21% in groups deficient in ALS [5]. Further research in ALSKO mice demonstrated that the glucose clearance rate was faster as compared with control mice [3, 4]. Deletion of ALS ameliorated the insulin resistance that develops in the IGF-1-deficient (LID) mice [3]. Interestingly, the glucose intolerance and hyperinsulinemia that were induced by GH treatment attenuated in ALSKO mice as compared with wild-type mice [28]. The mechanism is currently unclear but may be related to GH and IGF-1. If insuffient ALS to bind IGF-1 and IGFBP, the free form of IGF-1 will be increased and then suppression pituitary GH secretion via negative feedback. Studies have documented that insulin sensitivity increased in adults with GH deficiency [29]. In type 1 diabetic subjects with GH deficiency, the daily insulin requirement can be reduced, and these patients are more prone to episodes of hypoglycemia [30]. Furthermore, increased levels of free IGF-1 can enhance glucose uptake because IGF-1 has insulin-like functions [31].

RSG was demonstrated to improve insulin resistance in large clinical trials [8, 32]. Our results showed that HOMA-IR decreased at 12 weeks, but the improvement did not persist until the end of trial. We also observed that several unfavorable markers were elevated when diabetes was treated with RSG, including body weight, total cholesterol, and LDLc levels (Table 2). The increase of total cholesterol was mostly from LDLc. The baseline LDLc levels were 3.3 ± 1.1 mmol/L, which was higher than the recommended target level of 2.6 mmol/L for type 2 diabetes [33]. After 24 weeks of RSG treatment, there was a 27.6% increase of LDLc levels observed in our study. Rate of LDL > 2.6 mmol/L increased from 70.0% to 93.1% in RSG group. We did not find changes of HDL levels. This finding is consistent with the findings from previous clinical trials. HDL is minimally or nonsignificantly [8, 10, 34] elevated. Dyslipidemia after RSG treatment raised the concern of cardiovascular risks [35].

On the basis of the correlations between ALS, insulin sensitivity, and LDLc at baseline, we tested if RSG decreased ALS levels and if a change in ALS is beneficial. An inhibitory effect of RSG treatment on ALS levels was noted only in the subgroup of nonobese individuals with type 2 diabetes (Table 3). Why the effect of RSG treatment on ALS levels was dependent on BMI? The exact mechanism is not known. However, it has been previously reported that obese subjects exhibit blunted GH responses to insulin-induced hypoglycemia [24] or hyperglycemia [25] as compared to the nonobese individuals. This lack of feedback regulation on the GH-IGF1-ALS axis found in the obese subjects might explain our findings of the heterogeneous effect of RSG treatment on ALS levels observed in this study.

ALS was negatively correlated with LDLc levels at baseline (Table 4). After RSG treatment, we observed that ALS levels could predict changes in total cholesterol. Nissen and Wolski reported that RSG raised the risk of cardiovascular events. One hypothesis to explain this observation was the elevation of LDLc after RSG treatment [35]. Interestingly, ALS was recently studied as a cardiovascular biomarker [36], which is reasonable considering our results showing an inverse correlation between changes in ALS and LDLc during RSG therapy. Hypercholesterolemia has detrimental effects on insulin secretion and even causes islet cell apoptosis when oxidative LDLc is taken up via LDL receptors [37]. In an Asian population, LDLc levels were associated with insulin resistance [38, 39]. Although baseline serum ALS levels were negatively associated with HOMA2-%S, concurrent changes in total cholesterol levels diminished the benefit of decreased ALS on insulin sensitivity over time.

Despite the strengths of this study, such as the standardized methods used to collect the information and proper blood sample storage, there are certain limitations that need to be considered. First, the sample sizes were relatively small, which might severely reduce the statistical power for subgroup analyses according to BMI status. In the nonobese group, the total sample size was 19. A power analysis showed a 1-*β* value of only 0.55 (two-sided *α* = 0.05). Second, the covariates used in the multivariate regression models were assumed by baseline correlation analysis. A larger sample size will be needed for adjusting for more possible variables. Third, the levels of growth hormone, IGF-1, or the IGFBPs were not available to add to the model for a better understanding of the feedback regulation of RSG treatment on the GH-IGF1-ALS axis.

Our findings revealed a BMI-dependent effect of RSG treatment on ALS levels in individuals with type 2 diabetes. In nonobese diabetic subjects, serum ALS levels decreased after RSG treatment. Insulin sensitivity and LDLc were associated with ALS levels at baseline. The change in plasma ALS concentration predicted the change in total cholesterol concentration and correlated with the change in insulin sensitivity. Further studies will be required to clarify the effects of RSG on insulin sensitivity via the GH-IGF1-ALS axis and the mechanism of ALS influencing cholesterol metabolism.

## Figures and Tables

**Table 1:** Clinical characteristics of the study subjects at baseline.

	Placebo group	RSG group	*P*
*N*	31	30	
Age	59.4 ± 8.6	58.4 ± 10.0	0.668
Female (%)	58.1%	56.7%	1.000
Body weight (kg)	65.7 ± 9.0	65.9 ± 11.5	0.943
Body length (m)	1.59 ± 0.08	1.59 ± 0.09	0.932
BMI (kg/m^2^)	26.0 ± 3.2	25.9 ± 2.9	0.868
Systolic blood pressure, mmHg	136 ± 17	128 ± 14	0.037
Diastolic blood pressure, mmHg	81 ± 10	80 ± 9	0.053
HbA1c, (%)	9.92 ± 1.66	9.74 ± 1.36	0.646
Fasting plasma glucose, mmol/L	11.14 ± 3.00	10.95 ± 2.72	0.801
Fasting plasma insulin, pmol/L	94.55 ± 53.92	82.03 ± 42.23	0.391
Total cholesterol, mmol/L	5.80 ± 1.12	5.28 ± 0.92	0.055
Total triglyceride, mmol/L	2.42 ± 1.85	2.15 ± 1.25	0.583
LDLc, mmol/L	3.53 ± 1.22	3.13 ± 0.83	0.134
HDLc, mmol/L	1.15 ± 0.29	1.22 ± 0.42	0.450
HOMA2-%S	66.37 ± 41.49	66.70 ± 37.75	0.383
HOMA2-%B	41.52 ± 36.27	36.81 ± 39.01	0.631
HOMA-IR	6.28 ± 3.30	5.50 ± 3.18	0.368
HOMA-*β*	49.36 ± 50.09	43.53 ± 65.45	0.502
ALS, mU/mL	1284.9 ± 459.4	1474.9 ± 842.8	0.854

Each value represents the mean ± standard deviation. The *P* value is derived from a Student's *t*-test. RSG: rosiglitazone; BMI: body mass index; HbA1c: hemoglobin A1c; LDLc: low-density lipoprotein cholesterol; HDLc: high-density lipoprotein cholesterol; HOMA2-%S: homeostatic model assessment version 2 insulin sensitivity; HOMA2-%B: homeostatic model assessment version 2 *β*-cell function; HOMA-IR: homeostatic model assessment of insulin resistance; HOMA-*β*: homeostatic model assessment of *β*-cell function; ALS: acid-labile subunit.

**Table 2:** Changes in metabolic parameters after 24 weeks of treatment.

	12 weeks	24 weeks	*P*
*N*			
Placebo group	29	28	
RSG group	28	28	
Body weight (kg)			
Placebo group	−0.34 ± 1.33	−0.41 ± 1.21	<0.001
RSG group	1.12 ± 1.71	2.98 ± 2.09	
BMI (kg/m^2^)			
Placebo group	−0.13 ± 0.53	−0.16 ± 0.51	<0.001
RSG group	0.45 ± 0.73	1.21 ± 0.88	
Systolic blood pressure, mmHg			
Placebo group	−10.7 ± 15.3	−8.6 ± 18.8	0.083
RSG group	−0.4 ± 14.4	1.1 ± 11.7	
Diastolic blood pressure, mmHg			
Placebo group	−1.6 ± 9.4	−3.0 ± 8.3	0.901
RSG group	−1.2 ± 8.0	−0.6 ± 7.0	
HbA1c (%)			
Placebo group	−0.31 ± 1.43	−0.26 ± 1.37	<0.001
RSG group	−0.55 ± 1.08	−1.16 ± 1.09	
Fasting plasma glucose, mmol/L			
Placebo group	0.14 ± 3.25	0.75 ± 3.20	<0.001
RSG group	−1.47 ± 2.27	−1.42 ± 2.08	
Fasting plasma insulin, pmol/L			
Placebo group	−8.53 ± 29.11	−23.30 ± 36.67	0.505
RSG group	−19.96 ± 34.80	−10.79 ± 40.21	
Total cholesterol, mmol/L			
Placebo group	0.26 ± 0.85	0.20 ± 0.68	<0.001
RSG group	0.91 ± 1.01	0.85 ± 0.81	
Total triglyceride, mmol/L			
Placebo group	−0.28 ± 1.28	−0.22 ± 1.72	0.822
RSG group	−0.16 ± 1.10	−0.07 ± 0.60	
LDLc, mmol/L			
Placebo group	0.32 ± 1.03	0.31 ± 0.89	<0.001
RSG group	0.82 ± 0.97	0.86 ± 0.88	
HDLc, mmol/L			
Placebo group	0.05 ± 0.22	−0.005 ± 0.205	0.566
RSG group	0.08 ± 0.24	−0.006 ± 0.379	
HOMA2-%S			
Placebo group	6.92 ± 35.76	16.67 ± 22.60	0.533
RSG group	18.57 ± 15.48	11.40 ± 30.26	
HOMA2-%B			
Placebo group	−10.59 ± 29.84	−17.56 ± 36.40	0.011
RSG group	0.17 ± 42.02	2.58 ± 46.74	
HOMA-IR			
Placebo group	−0.19 ± 2.27	−0.99 ± 2.64	0.197
RSG group	−1.86 ± 2.28	−1.34 ± 2.40	
HOMA-*β*			
Placebo group	−16.27 ± 43.80	−26.40 ± 52.02	0.211
RSG group	−7.79 ± 69.26	−5.61 ± 77.65	
Acid-labile subunit, mU/mL			
Placebo group	21.99 ± 447.11	19.84 ± 395.67	0.627
RSG group	−88.17 ± 534.27	−117.16 ± 591.92	

Each value represents the mean ± standard deviation (*n*). *P* values represent the between-group comparisons of the changes in the 12- and 24-week values from the baseline value. RSG: rosiglitazone; BMI: body mass index; HbA1c: hemoglobin A1c; LDLc: low-density lipoprotein cholesterol; HDLc: high-density lipoprotein cholesterol; HOMA2-%S: homeostatic model assessment version 2 insulin sensitivity; HOMA2-%B: homeostatic model assessment version 2 *β*-cell function; HOMA-IR: homeostatic model assessment of insulin resistance; HOMA-*β*: homeostatic model assessment of *β*-cell function.

**Table 3:** Changes of serum ALS levels by treatment in subjects categorized as nonobese (BMI < 24 kg/m^2^) or obese (BMI ≥ 24 kg/m^2^).

	Nonobese subjects (BMI < 24 kg/m^2^)		Obese subjects (BMI ≥ 24 kg/m^2^)	
	Placebo	RSG	Placebo	RSG
*N*	10	9	21	21
Baseline ALS levels, mU/mL	1350.0 ± 574.2	1675.8 ± 1030.1	1253.9 ± 406.3	1388.8 ± 761.3
24-week ALS levels, mU/mL	1633.5 ± 582.6	1386.5 ± 627.3	1148.2 ± 439.8	1345.4 ± 593.6
Difference	283.5 ± 517.4	−289.2 ± 634.0*	−105.7 ± 251.1	−43.4 ± 572.9

*P = 0.0275 compared to the subjects in the placebo group. The *P* value is from the Wilcoxon rank-sum test. ALS: acid-labile subunit; BMI: body mass index; RSG: rosiglitazone.

**Table 4:** The correlations between HOMA2-%S and serum LDLc with ALS concentrations, analyzed with 4 linear regression models. Baseline serum ALS concentrations and change of ALS at 24 weeks were the dependent variables.

	Baseline ALS				Change of ALS at 24 weeks			
	HOMA2-%S	*P*	LDLc	*P*	Change in HOMA2-%S	*P*	Change in LDLc	*P*
Unadjusted	−0.262 ± 0.114	0.025	−0.156 ± 0.068	0.025	3.923 ± 2.711	0.156	−6.836 ± 77.616	0.930
Model 1: age, sex, and BMI	−0.165 ± 0.115	0.156	−0.165 ± 0.062	0.010	4.122 ± 2.832	0.154	−29.159 ± 80.044	0.718
Model 2: age, sex, BMI, and HbA1c	−0.166 ± 0.116	0.158	−0.171 ± 0.063	0.009	5.421 ± 2.789	0.060	−2.478 ± 80.444	0.976
Model 3: age, sex, BMI, HbA1c, and LDLc	−0.145 ± 0.111	0.196			5.819 ± 2.844	0.048		
Model 4: age, sex, BMI, HbA1c, and HOMA2-%S			−0.166 ± 0.064	0.012			−11.370 ± 80.531	0.889

Baseline levels of ALS and HOMA2-%S were log-transformed. ALS: acid-labile subunit; HbA1c: hemoglobin A1c; BMI: body mass index; HOMA2-%S: homeostatic model assessment version 2 insulin sensitivity; LDLc: low-density lipoprotein cholesterol.
